# Hashimoto’s Thyroiditis among Patients with Thyroid Disorders Visiting a Tertiary Care Centre

**DOI:** 10.31729/jnma.8236

**Published:** 2023-08-31

**Authors:** Suraj Parajuli, Navin Bhatt, Anil Regmi, Subodh Chapagain, Pradumna Panday, Arjan Singh, Shristi Nepal, Pragya Karki, Shweta Agrawal, Jyoti Bhattarai

**Affiliations:** 1 Department of Internal Medicine, Metro Kathmandu Hospital, Maharajgunj, Kathmandu, Nepal; 2 Nepal Medical College and Teaching Hospital, Jorpati, Kathmandu, Nepal; 3 Manosamajik Apanga Bandi Hospital, Nakkhu, Lalitpur, Nepal; 4 Everest Hospital, New Baneshwor, Kathmandu, Nepal

**Keywords:** *anti-thyroid peroxidase antibodies*, *Hashimoto's thyroiditis*, *thyroid disorders*

## Abstract

**Introduction:**

Hashimoto's thyroiditis is a chronic autoimmune lymphocytic thyroiditis characterised by thyroid autoantibodies. Early detection and treatment of this condition help in reducing the morbidity and mortality associated with it. The aim of the study was to find out the prevalence of Hashimoto's thyroiditis among patients with thyroid disorders visiting a tertiary care centre.

**Methods:**

A descriptive cross-sectional study was conducted among patients visiting the outpatient department of a tertiary care centre. Data from 14 April 2017 to 13 April 2019 was collected between 30 June 2022 to 15 September 2022 from medical records. Ethical approval was obtained from the Nepal Health Research Council. Hashimoto's thyroiditis was diagnosed based on clinical presentation and positive antibodies to thyroid antigens. Convenience sampling method was used. The point estimate was calculated at a 95% Confidence Interval.

**Results:**

Among 813 patients with thyroid disorders, 393 (48.33%) (44.89-51.77, 95% Confidence Interval) had Hashimoto's thyroiditis. The manifestation of the spectrum of Hashimoto's thyroiditis were euthyroid in 215 (54.70%), subclinical hypothyroidism in 102 (25.95%), subclinical hyperthyroidism in 23 (5.85%), overt hyperthyroidism in 9 (2.30%) and overt hypothyroidism in 4 (1.02%).

**Conclusions:**

The prevalence of Hashimoto's thyroiditis among patients with thyroid disorders was higher than in other studies done in similar settings.

## INTRODUCTION

Hashimoto's thyroiditis, also known as chronic lymphocytic or autoimmune thyroiditis is a chronic autoimmune thyroid disease characterised by thyroid autoantibodies, with presence of thyroid peroxidase antibodies, lymphocyte infiltraton and thyroglobulin antibodies.^1^ It is present in around 20-30% of patients with thyroid diseases.^2^ It is the most common cause of hypothyroidism, more common in women of age 30 to 50 years.^3^ Hashimoto's thyroiditis presents with goitre, fatigue, weight gain, hair loss, bradycardia, constipation, abnormal menstruation, difficulty concentrating.^4^

Many patients with Hashimoto's thyroiditis, even in the euthyroid state, have increased body weight, metabolic disorders, and reduced quality of life and is treated with levothyroxine.^5,6^ Therefore, close attention should be made towards the diagnosis and its management.

The aim of the study was to find out the prevalence of Hashimoto's thyroiditis among patients with thyroid disorders visiting a tertiary care centre.

## METHODS

A descriptive cross-sectional study was conducted among patients presenting to the Department of Internal Medicine of Metro Kathmandu Hospital, Maharajgunj, Kathmandu, Nepal. Data from 14 April 2017 to 13 April 2019 was collected between 30 June 2022 to 15 September 2022 from medical records. Voluntary consent was taken from the individual subjects during the outpatient visit. Ethical approval was obtained from the Nepal Health Research Council (Reference number: 4161). All patients who had clinical features and examination findings indicative of thyroid disorder and underwent thyroid function tests including anti-TPO antibodies were evaluated. For each patient who had completed both tests within the study duration, their data were considered as a sample unit for analysis. Patients with incomplete lab records or missing data in the database were excluded from the study. Convenience sampling method was used. The sample size was calculated by using the following formula:


$$\begin{array}{ll} \text{n} & ={\text{Z}}^{2}\times \frac{\text{p}\times \text{q}}{{\text{e}}^{\text{2}}}\\ & =1.96^{2}\times \frac{0.50\times 0.50}{0.04^{2}}\\ & =600 \end{array}$$

Where

n = minimum required sample size
Z = 1.96 at 95% Confidence Interval (CI)
p = prevalence taken as 50% for maximum size calculation
q = 1-p
e = margin of error, 4%

The calculated sample size was 600. However, 813 subjects were included in our study. Data were collected from the hospital records in a predetermined encrypted format covering the relevant variables of the study. Diagnosis of Hashimoto's thyroiditis was made on the basis of history, physical examination and laboratory findings which included thyroid autoantibodies, with presence of thyroid peroxidase antibodies, lymphocyte infiltraton and thyroglobulin antibodies.^1^ The data was entered into Microsoft Excel 2016 and analysed using IBM SPSS Statistics version 28.0. The point estimate was calculated at a 95% Confidence Interval.

## RESULTS

Among 813 patients with thyroid disorders, 393 (48.33%) (44.89-51.77, 95% Confidence Interval) had Hashimoto's thyroiditis. Among 393 patients, 54 (13.74%) were male and 339 (86.26%) were female.

Among 393 patients, 142 (50.35%) were in the age group 20-30 years followed by the age group of 31-40 years with 128 (32.57%) (Table 1).

**Table 1:** Age-wise distribution of study subjects (n= 393).

Age group (years)	n (%)
<20	19 (4.83)
20-30	142 (36.13)
31-40	128 (32.57)
41-50	65 (16.54)
51-60	26 (6.62)
>60	13 (3.31)

Among 393 patients, 215 (54.70%) were euthyroid (Table 2).

**Table 2:** Different thyroid disorders in the study subjects (n= 393).

Thyroid disorders	n (%)
Euthyroid	215 (54.70)
Hypothyroidism	9 (2.30)
Subclinical hypothyroidism	23 (5.85)
Subclinical hyperthyroidism	4 (1.02)
Hyperthyroidism	102 (25.95)
Others	40 (10.18)

## DISCUSSION

Among 813 patients with thyroid disorders, 393 (48.33%) had Hashimoto's thyroiditis. This finding is higher compared to the study based in different cities in India, where the prevalence of anti-TPO antibodies was found to be 21.85%.^7^ Another similar study done in Greece showed the prevalence to be 30.4%.^8^ This may signify that anti-TPO antibody-positive thyroid disorder is more prevalent among the people of Nepal.

In our study, 54 (13.74%) were male and 339 (86.26%) were female. This is in accordance with the fact that thyroid disorders are more prevalent in females and thus, tested more for the diagnosis of thyroid disorders. Also, the prevalence of thyroid autoimmunity is significantly greater in women.^9^

According to our study, the maximum 142 (36.13%) subjects with positive anti-TPO antibody tests were from 20 to 30 years. This age is very low when compared to other studies which usually mention it to be common in 30-50 years.^10^ This remains an area of study which can be explored further.

Among the patients with positive anti-TPO antibodies, euthyroidism was present in 215 (54.70%) and subclinical hypothyroidism was present in 23 (5.85%). This signifies that even though the patients are found to be positive for anti-TPO antibodies, the majority of them may have normal thyroid function test results. This could have been because the tests might have been done on the patients after they had been initiated with treatment for their thyroid disorders. It should be noted that anti-TPO may manifest before or after a lab test reveals a thyroid hormone test abnormality.^11^ Thereby, combined testing of anti-TPO and TFT is helpful in identifying individuals at potential risk. This would prevent long-term morbidity since anti-TPO antibody is related to future overt hypothyroidism and other autoimmune diseases. So, regular follow-ups with serial thyroid function tests to identify changes in thyroid hormone levels in patients with positive anti-TPO antibodies would ensure the early identification of thyroid disorders in these patients. In addition, some studies have demonstrated that early treatment of thyroid dysfunction, mainly subclinical hypothyroidism, can prevent the development of overt hypothyroidism and associated morbidity.^12^ Therefore, our study attempted to demonstrate the importance of performing anti-TPO tests along with thyroid hormone levels in clinical practice.

Since the study is based on a single centre, the findings cannot be generalised to a greater level. Also, there has been no assessment of co-morbid conditions and associated risk behaviours such as diabetes, depression, and smoking due to the limitations of the study design.

## CONCLUSIONS

The prevalence of Hashimoto's thyroiditis among patients with thyroid disorders was higher than in other studies done in similar settings. Understanding the prevalence and clinical associations of Hashimoto's thyroiditis can assist healthcare professionals in providing optimal management and care for affected individuals.
